# Methods for telomere length measurement: an update on current technologies and emerging approaches

**DOI:** 10.3389/fmolb.2025.1725112

**Published:** 2025-12-18

**Authors:** Julia A. Makarova, Uliana D. Belova, Maria I. Zvereva, Maxim Yu. Shkurnikov, Alexander G. Tonevitsky

**Affiliations:** 1 Faculty of Biology and Biotechnology, HSE University, Moscow, Russia; 2 Faculty of Chemistry, Lomonosov Moscow State University, Moscow, Russia

**Keywords:** telomeres, telomere length measurement, terminal restriction fragment analysis, MM-qPCR, Q-FISH, long-read telomere sequencing

## Abstract

Telomeres are nucleoprotein complexes at chromosome ends, composed of tandemly repeated specific DNA sequences along with associated proteins. In somatic cells, telomeres progressively shorten with each cell division, making telomere length a key biomarker of cellular aging. Moreover, alterations in telomeric attrition are characteristic of numerous lifestyle factors, age-related diseases, and cancers, establishing telomere length as both a pivotal biomarker and a central focus in contemporary biomedical research. Strong interest in this area drives the continuous development of new methods for telomere length measurement and improvements to existing ones. Currently, over two dozen such methods have been developed, making the ability to select the most appropriate one essential for addressing specific research objectives. This review provides a state-of-the-art survey of all existing methods, highlighting their advantages, limitations, and applications. Special attention is focused on the rapidly evolving field of adapting long-read sequencing technologies to enhance the efficiency of telomere length measurement, along with novel insights into the structure and diversity of telomeric sequences uncovered by this approach.

## 1 Introduction

Telomeres are specialized structures localized at the ends of chromosomes. They consist of short tandemly repeated DNA sequences (TTAGGG in vertebrates (Meyne et al., 1989)) and, in humans, typically range from 5 to 15 kilobases (kb) in length. Together with associated proteins, telomeres protect chromosome termini from end-to-end fusions and prevent essential genes from degradation during DNA replication cycles (Jones-Weinert et al., 2025).

**FIGURE 1:**
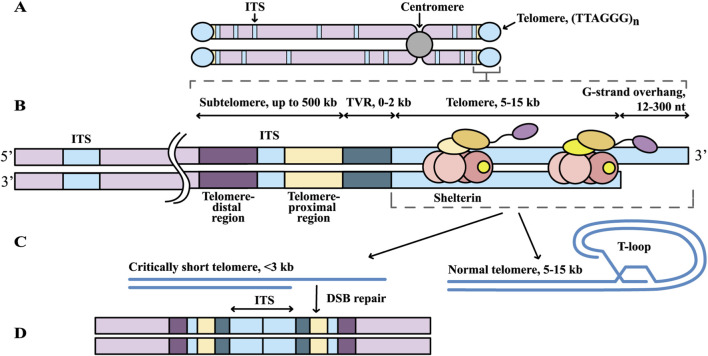
Structure of human telomeres. **(A)** Localization of telomeric repeats on the chromosome (shown in light blue). **(B)** Structure of the subtelomeric and telomeric regions of human chromosomes. Shelterin complexes bound to telomeric repeats are shown. **(C)** Comparison of normal and critically shortened telomeres: when their length falls below 3 kb, the protective T-loop structure is lost. **(D)** Formation of an ITS may result from double-strand break (DSB) repair involving critically shortened telomeres.

At their proximal end, telomeres are flanked by subtelomeres that exhibit substantial variation in size and structure across chromosomes and individuals (Kwapisz and Morillon, 2020), with lengths extending up to 500 kb (Riethman, 2009; Young et al., 2020) (Figures 1A,B). These regions consist of segmental duplications and repetitive elements that are shared among multiple subtelomeres, together with sequences that are unique to individual chromosome ends. Subtelomeres can be divided into two domains. The proximal domain, located next to the telomere, is enriched in repeats (TAR1 and other), largely lacks genes, and is conserved across many chromosome ends (Kwapisz and Morillon, 2020). In contrast, the distal domain is more heterogeneous and contains both sequences shared among multiple chromosome termini and sequences specific to a given subtelomeric region, as well as genes that are nonessential for cell viability (Kwapisz and Morillon, 2020). These two domains are often separated by telomeric repeat regions known as interstitial telomeric sequences (ITSs) (Figures 1A,B). ITSs are found not only in subtelomeric regions, but also in internal regions of the genome (Shubernetskaya et al., 2017). In the human genome, they are predominantly short arrays of 2–25 TTAGGG repeats, with several thousand copies reported (Aksenova and Mirkin, 2019); longer ITSs are much less common. According to the telomere-to-telomere (T2T) CHM13 assembly, only 79 ITSs longer than 200 bp have been identified (Hsieh et al., 2025). Thus, on average, ITSs account for about 5% of the total telomere length (TL) in the genome and therefore do not significantly contribute to TL measurement results.

At the junction between the telomere and subtelomere lies the telomere variant repeat (TVR) region. This region consists of degenerate telomeric repeats that typically differ from the canonical sequence by a single nucleotide substitution, insertion, or deletion (Figure 1B) (Allshire et al., 1989). On most chromosome arms, TVR regions span 0–2 kb, though in some alleles they may extend up to ∼8 kb (Tham et al., 2023; Stephens and Kocher, 2024). Due to their reduced affinity for telomere-binding proteins (Broccoli et al., 1997; Nishikawa et al., 2001; Conomos et al., 2012), TVRs generally lack canonical telomeric functions. Importantly, nearly every chromosome arm harbors a unique pattern of TVR region (Stephens and Kocher, 2024), which facilitates assignment of individual telomeres to their specific chromosomal arms in sequencing data.

Telomeres terminate with a 3′single-stranded overhang of 130–230 nucleotides (Makarov et al., 1997), always formed by the G-rich strand (Figure 1B). This structure facilitates the formation of the so-called T-loop, in which the chromosomal end is embedded within the telomeric sequence (Figure 1C) (Griffith et al., 1999). T-loop size correlates positively with TL (Wang et al., 2004). The six-subunit shelterin complex binds to this region and prevents it from being recognized as a DNA double-strand break by cellular repair machinery (O’Sullivan and Karlseder, 2010). During each cell division, telomeres shorten by approximately 50–200 bp (Cai and Lange, 2023). Once a critical length is reached, telomeres lose their protective capacity, likely due to the inability to form T-loops and the reduced binding affinity of shelterin proteins (Ruis and Boulton, 2021). The threshold for “critically short” telomeres varies but is generally in the range of 1–3 kb (Lai et al., 2023; Smoom et al., 2025). When telomeres become critically short, the chromosome end is perceived as a DNA break, triggering a damage response through ATM and/or ATR kinases (Doksani, 2019), which activate signaling cascades including phosphorylation of p53 (Fagagna et al., 2003). This leads to chromosome fusion Figure 1D), persistent cell cycle arrest, activation of the apoptotic pathways, or transition to replicative senescence—one of the key mechanisms of cellular aging, when the cell remains metabolically active but ceases to proliferate (Deng et al., 2008; Yegorov, 2023). When functioning properly, these processes prevent malignant transformation. However, the accumulation of senescent cells in tissues impairs their function and contributes to aging and age-related diseases. Telomere attrition is also induced by oxidative stress and may be more pronounced than losses during cell division (von Zglinicki, 2002); it can occur even in non-dividing cells (Gavia-García et al., 2021). Oxidative stress, in turn, can be triggered by chronic inflammation and various lifestyle factors, including chronic stress, sleep deprivation, smoking, and others (Sharifi-Rad et al., 2020; Davinelli et al., 2024).

Telomere attrition can be counterbalanced by telomerase, an enzyme that synthesizes new telomeric repeats. However, telomerase activity is limited in humans: it is predominantly maintained in germline, stem, and certain immune cells, while in most somatic tissues, telomerase activity is suppressed (Zvereva et al., 2010). In cancer cells, telomeres are maintained either through telomerase reactivation or by alternative TL maintenance mechanisms (ALT) (Kim et al., 1994; O’Sullivan and Greenberg, 2025), which represent a recombination-based telomerase-independent pathway enabling some cancer cells to restore TL and acquire replicative immortality. Importantly, oxidative stress-induced telomere shortening can be slowed or even reversed *in vitro* by antioxidants, opening promising therapeutic perspectives (Furumoto et al., 1998; Prasad et al., 2017). Recent *in vivo* studies have shown that the strength of the correlation between TL and oxidative stress depends on the choice of TL measurement method, highlighting the importance of developing accurate TL assays (Armstrong and Boonekamp, 2023).

TL is highly heterogeneous in human cells (Demanelis et al., 2020). The longest telomeres are found in testes, reflecting high telomerase activity in spermatogenic cells (Aston et al., 2012). Skeletal muscle and adipose tissue also exhibit relatively long telomeres, likely due to their low proliferative activity (Demanelis et al., 2020). In contrast, tissues with high proliferative activity, such as skin and blood, have shorter telomeres (Demanelis et al., 2020). Among leukocytes, B-cells harbor the longest telomeres, whereas CD8^+^CD28^−^ cytotoxic T-lymphocytes carry the shortest (Lin et al., 2016). Moreover, the rate of telomere shortening varies by cell type: in B cells telomeres are shortened most rapidly (∼196 bp/year), followed by CD8^+^CD28^−^ T-сells (∼90 bp/year), while telomeres in other lymphocyte subsets shorten more slowly (Lin et al., 2016). This apparent discrepancy—longer telomeres but faster attrition in B cells versus shorter telomeres but slower attrition in CD8^+^CD28^−^ T cells—likely reflects a combination of regression to the mean (Verhulst et al., 2013) and differential exposure to physiological stressors across cell types (Lin et al., 2016). At the chromosomal level, differences in TL are observed between arms (e.g., 17p is short, 4q is long) (Martens et al., 1998; Karimian et al., 2024), as well as between maternal and paternal homologs (Karimian et al., 2024).

**FIGURE 2:**
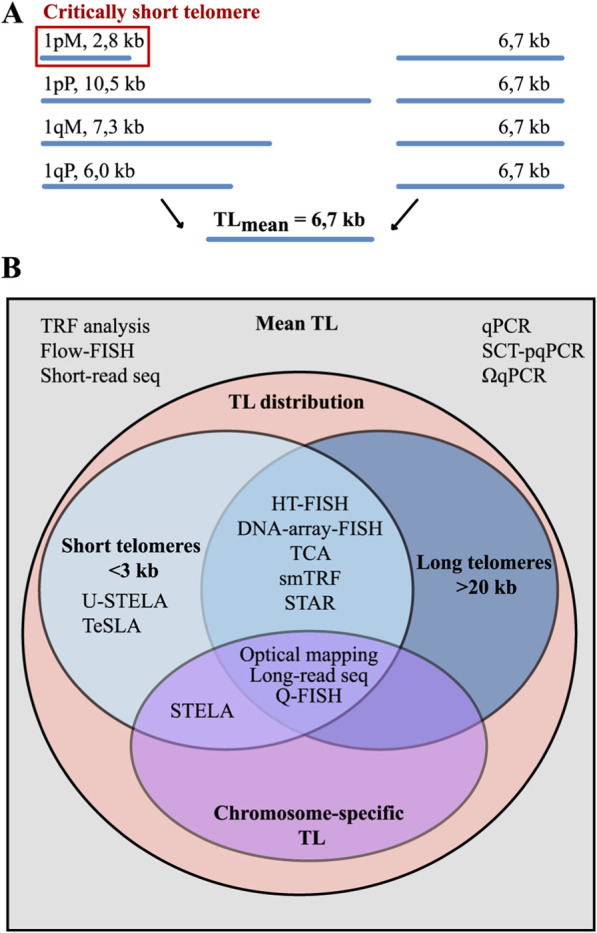
TL heterogeneity and methods for its assessment. **(A)** The averaging of TL across all chromosomes can mask significant intra- and inter-chromosomal variations, such as the presence of critically short or excessively long telomeres. **(B)** The analytical capabilities of TL measurement methods vary significantly in the depth of analysis they provide. They can be broadly classified in two groups. Methods for average TL estimation provide only the mean TL across all chromosomes. Methods for TL distribution analysis is a more advanced group that yields a pattern of TL in a sample, allowing for the quantification of critically short (<3 kb) and excessively long telomeres. The most advanced methods, such as long-read sequencing, allow for the determination of chromosome arm-specific TL.

The variation in TL among chromosomes highlights the importance of assessing not only changes in mean TL but also the critical shortening of individual telomeres (Figure 2A). Their detection is important because it is precisely the critically short telomeres that trigger chromosome fusion, senescence and apoptosis (Abdallah et al., 2009; Hemann et al., 2001). These telomeres can contribute to the formation of specific cytogenetic abnormalities or modulate the expression of genes located both in regions adjacent to telomeres and at a distance from telomeres due to the so-called telomeric position effect—the phenomenon by which TL influences transcriptional activity of certain genes (Robin et al., 2014).

Although for most diseases no direct association has yet been identified between telomere shortening on a specific chromosome and the development of a specific clinical phenotype, in some pathologies, such as leukaemia, chromosome-specific changes in the telomere profile are observed in addition to general telomere shortening. For example, in chronic myeloid leukaemia, the longest telomeres are often located on 18p and Xp, and the shortest on 21p and 21q, whereas in normal conditions the situation differs: the maximum TL is observed on 5p, 3p and 4p, and the minimum on 19p, 17p and 20q (Samassekou et al., 2009). Therefore, approaches based on the analysis of so-called telomeric signatures, which take into account the variability of TL across all chromosomes, have recently emerged (Toupance et al., 2019; Shah and Sethuraman, 2025). A predictive machine learning model based on telomeric signatures has been shown to distinguish between tumor and normal samples with a high degree of accuracy (Shah and Sethuraman, 2025).

Recognition that TL is associated with ageing, age-related diseases, genomic instability and neoplastic transformation (Lin et al., 2010; Gruber et al., 2021; Yegorov et al., 2021; Borges et al., 2022), together with direct demonstration that a single critically short telomere can arrest cell division and trigger senescence (Abdallah et al., 2009), sparked a surge of interest in this field. However, measuring TL is quite a challenging task. The significant length and short tandem repeat composition of telomeres make direct implementation of conventional PCR and short-read sequencing approaches inapplicable. Furthermore, due to the TL heterogeneity, methods that provide only mean TL fail to detect essential features, such as the presence of critically short telomeres (Figure 2A).

To date, approximately 20 methodological approaches have been developed for measuring TL, none of which is universal, each with its own advantages and limitations. The choice depends on the goal of the study, and this review aims to help researchers select the appropriate method by providing an extensive survey of all TL measurement techniques, including the latest methodological updates and bioinformatic tools. The most common methods are qPCR, specially designed for TL measurement (Cawthon, 2002; 2009; Nettle et al., 2021), and Southern blot analysis of terminal restriction fragments (TRF) (Kimura et al., 2010b). However, these methods allow the measurement of only mean TL values. To overcome this, high-resolution techniques have been developed to examine TL heterogeneity. These include hybridization-based Quantitative Fluorescence *In Situ* Hybridization (Q-FISH) (Canela et al., 2007) as well as qPCR-based approaches: Single Telomere Length Analysis (STELA) (Baird et al., 2003) and its derivatives Universal STELA (U-STELA) (Bendix et al., 2010) and Telomere Shortest Length Assay (TeSLA) (Lai et al., 2017) (Figure 2B). Long-read sequencing offers advanced capabilities for analyzing chromosome-specific telomeric sequences, and this review highlights recent progress in this rapidly evolving field. The methods for TL measurement are classified by their underlying approaches: (1) hybridization-based, (2) combined hybridization and PCR, (3) PCR-only, and (4) sequencing with short or long reads. For each method, the advantages, limitations and other features are described. Key output metrics for each method, as well as the time and DNA amount required for analysis are summarised in Table 1.

**TABLE 1:** Key features of TL measurement methods.

Method	Data output and resolving power	Required amount of DNA/cells	Correlation with TRF (*R* ^2^)	Reproducibility(intra- and inter-assay CV)	Number of samples processed simultaneously	Experiment duration excluding DNA extraction time	Throughput	Resolution, bp	Frequency of use	References
TRF	Mean TL in a bulk cell population	>1 µg DNA	Gold standard	0.2%–4.6% (intra) 1.5%–15.0% (inter)	30 (on one gel)	3–4 days	Medium	1,000	High	Kimura et al. (2010b), Aviv et al. (2011), Aubert et al. (2012b), Gutierrez-Rodrigues et al. (2014), Martin-Ruiz et al. (2015), Mender and Shay (2015)
Metaphase Q-FISH	Single-cell chromosome-specific TL	10–20 cells	0.90	12.4% (intra)	5	2–3 days	Low	300	Medium	Lansdorp (1996), Poon et al. (1999), Slijepcevic (2001), Zheng et al. (2009), Vera and Blasco (2012)
HT Q-FISH	Single-molecule TL in a bulk cell population	10^5^ cells	0.99	<5.0% (intra)	96–384 (on 96- or 384-well plates)	2–3 days	High	ND	Medium	Canela et al. (2007), de Pedro et al. (2020)
Flow-FISH	Mean TL in a bulk cell population	10^5^ cells	0.51–0.60	2.2%–10.8% (intra) 2.5%–9.5% (inter)	96	2–3 days	High	300	Medium	Baerlocher et al. (2006), Gutierrez-Rodrigues et al. (2014), Alder et al. (2018)
DNA-array-FISH	Single-molecule TL in a bulk cell population	0,5 µg DNA	0.76–0.98	3.2%–4.0% (intra) 1.4%–3.4% (inter)	96–384 (on 96- or 384-well plates)	2–3 days	High	ND	Low	Zheng et al. (2024)
Optical mapping	Chromosome-specific TL in a bulk cell population	0,3 µg DNA	ND	14.6% (inter)	10	2–3 days	Low	ND	Low	Uppuluri et al. (2021)
TCA	Single-molecule TL in a bulk cell population	10^6^ cells (>2 µg DNA)	0,80	ND	10	3 days	Low	ND	Low	Kahl et al. (2020)
smTRF	Single-molecule TL in a bulk cell population	0,5 µg DNA	0.94	ND	10	1–2 days	Low	∼ few nucleotides	Low	Gao et al. (2025)
STELA	Chromosome-specific TL for several chromosomes in a bulk cell population	10–50 ng DNA	NA	3% (intra) 5% (inter)	30 (on one gel)	3–4 days	Medium	100	Low	Baird et al. (2003), Britt-Compton et al. (2006)
U-STELA	Single-molecule TL in a bulk cell population	10–50 ng DNA	NA	5% (inter)	30 (on one gel)	3–4 days	Medium	100	Low	Bendix et al. (2010)
TeSLA	Single-molecule TL in a bulk cell population	10–50 ng DNA	NA	4.3% (intra) 1.6%–3.9% (inter)	30 (on one gel)	3–4 days	Medium	100	Low	Lai et al. (2017)
Singleplex qPCR	Mean relative TL (T/S ratio) in a bulk cell population	10–50 ng DNA	0.20–0.68	9.5%–19% (intra) 6.5%–28% (inter)	96–384 (on 96- or 384-well plates)	<3 h	High	NA	High	Aviv et al. (2011), Shen et al. (2011), Gutierrez-Rodrigues et al. (2014)
MM-qPCR	Mean relative TL (T/S ratio) in a bulk cell population	10–50 ng DNA	0.84	<10% (intra) <5% (inter)	96–384 (on 96- or 384-well plates)	<3 h	High	NA	High	Cawthon, 2009; Gao et al. (2025)
qPCR with a calibration standard	Mean TL in a bulk cell population	10–50 ng DNA	0.77	2% (intra) 7% (inter)	96–384 (on 96- or 384-well plates)	<3 h	High	NA	Medium	O’Callaghan et al. (2008)
STAR	Single-molecule TL in a bulk cell population	<1 ng DNA	0.96	ND	48 (on one chip)	<3 h	High	200	Low	Luo et al. (2020)
SCT-pqPCR	Mean relative TL (T/S ratio) in single cells	10 pg DNA (single cell)	0.89	ND	96–384 (on 96- or 384-well plates)	4–6 h	High	ND	Low	Wang et al. (2013)
ΩqPCR	Mean TL in single cells	10 pg DNA (single cell)	ND	ND	96–384 (on 96- or 384-well plates)	4–6 h	High	∼few nucleotides	Low	Xiong and Frasch (2021)
NGS	Mean TL/telomere content in a bulk cell population	>2 µg DNA	0.17–0.59	3.2% (intra) for TelSeq	from a few to up to ∼50 genomes in a single run	3–4 days	Medium	ND	Low	Ding et al. (2014), Nersisyan and Arakelyan (2015), Farmery et al. (2018), Feuerbach et al. (2019)
Long-read sequencing	Chromosome-specific TL in a bulk cell population	>2 µg DNA	0.79 (DTM)	Telomere Profiling 1.3% (intra) 2.3% (inter)	10 (when samples are multiplexed and enriched for telomeres)	3–4 days	Low	∼ few nucleotides	Low	Sholes et al. (2022), Karimian et al. (2024), Sanchez et al. (2024)

NA, not applicable; ND, Not determined.

The application of TL measurement techniques has already revealed an association between telomere attrition and a wide range of diseases and disorders. Mutations in telomerase genes (TERT, TERC) or other components of the telomere maintenance machinery (Armanios, 2009; Du et al., 2009; Diaz de Leon et al., 2010) cause telomeropathies, a group of hereditary syndromes that include pulmonary fibrosis, congenital dyskeratosis, bone marrow aplasia, and others. However, most diseases linked to TL are age-related and include, for example, cardiovascular (Xu et al., 2020), autoimmune (Wei et al., 2023; Ibraheem Shelash Al-Hawary et al., 2024), metabolic (Hemann et al., 2001), neurodegenerative disorders (Levstek et al., 2020) and cancer (Nersisyan et al., 2019). TL is also influenced by environmental and lifestyle factors, and favorable conditions are associated with telomere elongation, improved quality of life, and enhanced longevity (Epel et al., 2004; Astuti et al., 2017; Avetyan et al., 2019; Andreu-Sánchez et al., 2022). Although many studies have shown links between telomere shortening and disease or lifestyle, it is often unclear whether telomere shortening directly causes these diseases or is just associated with other factors. Recent advancements in TL measurement techniques hold the promise to address this question.

## 2 Hybridization-based methods

### 2.1 Terminal restriction fragment (TRF) analysis

TRF analysis was the first method developed for TL measurement in 1990 (Harley et al., 1990). It involves digesting genomic DNA with restriction endonucleases that do not cleave telomeric repeats followed by Southern blotting. It remains the “gold standard” for TL measurement. For maximum degradation of non-telomeric DNA, a combination of endonucleases is used, usually one of four sets: *Hinf*I/*Rsa*I [most frequently used (Kimura et al., 2010b)], *Hph*I/*Mnl*I, *Mse*I/*Nde*I, or *Bfa*I/*Cvi*AII/*Mse*I/*Nde*I. Although none of these enzymes cleave telomeres, each has its own pattern of cleavage of the subtelomeric region. For example, the endonucleases *Hph*I and *Mnl*I cleave DNA closer to the telomeric boundary, while *Hinf*I and *Rsa*I leave a longer subtelomeric sequence. As a result, fragments obtained using *Hinf*I/*Rsa*I are on average approximately 1 kb longer than those obtained using *Hph*I/*Mnl*I (Baird et al., 2006). Importantly, the TRF fragments include both telomeric and adjacent subtelomeric DNA, which systematically leads to overestimation of TL (Baird et al., 2006) and complicates direct comparisons with results from other TL measurement methods (Lai et al., 2018). Nevertheless, within the same cell type, the systematic overestimation of TL is uniform across samples and therefore does not confound the observed differences in TL.

Following digestion, DNA fragments are separated by gel electrophoresis, transferred onto nitrocellulose or nylon membranes, and hybridized with labeled probes complementary to telomeric repeats. While any telomeric strand can serve as a probe, sensitivity is often higher when using the G-rich strand (Herbert et al., 2003). Probes are typically labeled with digoxigenin (DIG) (Kimura et al., 2010b) or radioactive isotopes ([γ-32P] ATP or [α-32P]ATP (Mender and Shay, 2015; Jenkins et al., 2017)), providing high sensitivity. Average TL are determined by analyzing the distribution of probe hybridization signals across the gel relative to molecular weight markers, commonly combining a commercial marker (∼0.5–12 kb) and λ phage DNA fragments digested with *Hind*III (1.25–23.1 kb). TRF blot images provide an excellent illustration of the heterogeneity of TL within cell populations (see representative examples in (Kimura et al., 2010b; Karimian et al., 2024)). Several modifications of the TRF protocol have been introduced, notably in-gel hybridization that eliminates the need for membrane transfer. In this method, the gel is dried, DNA is denatured, and hybridization occurs directly within the gel using a labeled oligonucleotide probe. This streamlined approach simplifies the traditional TRF workflow, reduces hands-on time, and minimizes loss of telomeric DNA during the blotting process (Mender and Shay, 2015; Jenkins et al., 2017).

The TRF analysis provides a quantitative estimate (in kb) of the average TL across a cell population but does not allow TL measurement at the level of individual cells or chromosomes. A significant practical limitation is the need for a relatively large amount of DNA—from 1 to 5 μg per sample. The method may also have limitations for alleles with large TVR regions: the standard subtraction of the constant (2–4 kb) between the enzyme restriction site and the telomere boundary may be insufficient, potentially leading to overestimation of TL (Aubert et al., 2012b). In addition, the method cannot reliably distinguish the shortest and longest telomeres, as the presence of corresponding hybridization signals may result from restriction enzyme artifacts. Moreover, short telomeres hybridize with fewer probes, producing a disproportionately weak signal relative to their abundance, while longer telomeres generate a stronger signal due to hybridization with a larger number of probes (Kimura et al., 2010b).

This limitation led to the development of specialized software for analyzing TRF results: Telometric (Grant et al., 2001), TeloTool (Göhring et al., 2014) and WALTER (Web-based Analyser of the Length of Telomeres) (Lyčka et al., 2021), which outperform generic image analysis software (e.g., ImageQuant™ (Cytiva) and ImageJ). Telometric averages signal intensity across the entire gel lane assuming a normal distribution of TL, but does not verify this assumption. In practice, TL distribution is often asymmetrical, so this assumption is inaccurate and can lead to biased results that usually overestimate average TL (Göhring et al., 2014). To account for the peculiarities of hybridization probe signal formation, Telometric normalizes the signal intensity by dividing it by the number of available probe-binding sites. However, this correction may underestimate the average length of long telomeres (for example, 12 kb telomeres may be underestimated by 2 kb) because the probes may not fully saturate all available binding sites. TeloTool addresses this issue by fitting each gel lane profile with a Gaussian curve to calculate the mean, median, standard deviation, and goodness-of-fit, which assesses the suitability of the normal distribution assumption. It also corrects for signal intensity biases caused by probe hybridization variability and gel artifacts. However, in some cases, TeloTool may overestimate TL, especially when the Gaussian curve fit is poor. Importantly, this tool can detect atypical TL distributions, such as clearly bimodal patterns with distinct populations of short and long telomeres observed in certain telomeropathies (Norris et al., 2021) and cancers (Luo et al., 2020). It should be noted that since TeloTool uses Gaussian approximation, the quantitative assessment of complex bimodal distributions may be incorrect, and for their detailed analysis, it is preferable to use the recently developed WALTER tool (Lyčka et al., 2021). WALTER analyzes smoothed raw signals without assuming any specific distributional form. It automatically identifies telomeric regions and applies statistical procedures, including median and quartile estimations, while visualizing TL distributions. Current evidence suggests that WALTER provides the most balanced and reliable analysis of TRF results, particularly for atypical telomere profiles, whereas Telometric and TeloTool tend to overestimate TL.

### 2.2 Fluorescence *in situ* hybridization (FISH)

*In situ* hybridization methods employ fluorescently labeled peptide nucleic acid (PNA) probes, which are synthetic DNA analogs featuring a neutral peptide backbone instead of the conventional phosphate-sugar structure. This neutral backbone enhances stability by eliminating electrostatic repulsion, thereby improving hybridization efficiency and specificity. Moreover, PNA–DNA hybridization exhibits markedly greater sensitivity to base mismatches compared to DNA–DNA hybridization, ensuring high specificity of PNA probes. These probes also efficiently hybridize to single-stranded DNA regions even under low ionic strength conditions, enabling effective application in whole-cell assays (Narath et al., 2005). Presently, quantitative fluorescence *in situ* hybridization (Q-FISH), high-throughput Q-FISH (HT Q-FISH), flow-FISH, and DNA-array-FISH are employed for TL analysis (Li et al., 2025).

#### 2.2.1 Q-FISH and HT Q-FISH

One of the key advancements in fluorescence *in situ* hybridization-based methods is Q-FISH, which employs fluorescence microscopy to visualize telomeres and accurately quantify their length. Q-FISH is primarily performed in two formats: on metaphase (Poon et al., 1999) or interphase (Narath et al., 2005) nuclei. Telomeres are detected using specific PNA probes, while the non-telomeric chromatin is counterstained with DNA dyes such as DAPI or propidium iodide. This combination enables the identification of individual chromosomes and the localization of their telomeric ends. Whereas the TRF method measures average TL at all chromosomal ends collectively, this technique assesses the length of each of the 92 telomeres separately, providing high-resolution (∼0.2–0.3 kb) measurements (Lansdorp, 1996; Poon et al., 1999) and facilitating identification of critically short telomeres (Slijepcevic, 2001). Q-FISH requires rigorous calibration to derive absolute TL. Fluorescent signal intensities must be converted using standards such as plasmids containing known numbers of telomeric repeats. Additionally, fluorescent microspheres of defined size are used simultaneously to correct for fluctuations in light source intensity and imaging conditions. (Slijepcevic, 2001). An important limitation of Q-FISH is its low throughput. Furthermore, metaphase Q-FISH can only be applied to actively dividing cells, as it requires metaphase chromosome preparations, thereby restricting its broader applicability (Canela et al., 2007).

These limitations have been overcome by HT Q-FISH, which was specially designed to improve TL measurement and employs fluorescently labeled PNA probes hybridized to telomeric repeats within the nuclei of interphase cells cultured in 96-well plates (Canela et al., 2007). Following hybridization, automated fluorescence microscopy quantifies the telomeric signal in individual nuclei, enabling rapid analysis (one plate within approximately 2 hours) across thousands of cells. This approach facilitates the construction of TL distribution profiles and the reliable detection of cells harboring critically short telomeres. HT Q-FISH has demonstrated high reproducibility (intra-laboratory coefficient of variation <5%) and has been validated for large-scale epidemiological and clinical studies (Canela et al., 2007), including CLIA-certified testing in the United States.

#### 2.2.2 Flow-FISH

The flow-FISH method utilizes cell suspensions, which substantially enhances throughput. This technique relies on the hybridization of cells with fluorescently labeled PNA probes, followed by analysis through flow cytometry.

Each assay incorporates two types of control cells: an internal control, which reduces error in TL estimation, and a reference sample, which allows the conversion of fluorescence intensity into absolute TL values in kb. Bovine thymocytes are typically employed as the internal control, as they are readily available, smaller in size than human lymphocytes and granulocytes, and possess longer telomeres (15–20 kb). These cells are added to each tube containing the sample and analyzed concurrently by flow cytometry. Bovine thymocytes can be distinguished from human lymphocytes and granulocytes based on forward scatter parameters, which reflect cell size, and by the intensity of staining with a nonspecific DNA dye, which is influenced by DNA accessibility and therefore varies among cell types. Human cells with predetermined TL (for example, granulocytes with TL assessed by TRF analysis) serve as the reference sample.

Following hybridization and washing, cells are counterstained with a DNA dye and, optionally, with antibodies against surface markers, allowing identification of subpopulations such as granulocytes, T and B lymphocytes, and NK cells. The resulting cell suspension is analyzed by flow cytometry, recording the fluorescence intensity of each cell, which is directly proportional to the number of telomeric repeats. To account for autofluorescence, a parallel sample lacking the PNA probe is measured. TL is calculated by normalizing the fluorescence signal of the target cells to that of the internal control and calibrating this ratio using a reference sample with known TL, allowing conversion into absolute values. This method yields average TL estimates for an entire cell population (Hultdin et al., 1998). Flow-FISH has been successfully employed for diagnosing inherited and acquired telomere maintenance disorders (Alder et al., 2018), as well as for monitoring hematopoietic status after bone marrow transplantation (Rufer et al., 2001).

The main drawbacks of the method are associated with the need to use unfixed cells, which are fragile under FISH conditions. Some protocols use fixatives such as formaldehyde to ensure structural stability of cells in suspension and to preserve epitopes or surface-bound antibodies. However, these reagents can unpredictably impair probe hybridization. Even low concentrations of crosslinking agents have been shown to reduce fluorescence signal and increase variability (Baerlocher et al., 2006). Moreover, to minimize nonspecific binding of PNA probes to cytoplasmic structures and reduce background signal caused by cytoplasmic autofluorescence, the use of isolated nuclei rather than whole cells has been proposed (Wieser et al., 2006). Flow-FISH may also be unsuitable for certain cell types and is most often applied to fresh blood samples (Aubert et al., 2012a). As with Q-FISH, the method requires rigorous calibration.

#### 2.2.3 DNA-array-FISH

Recently, the FISH method was integrated with DNA microarray technology (Zheng et al., 2024). Unlike the previously described FISH-based approaches, this method employs purified genomic DNA, digested with restriction endonucleases that do not cleave telomeric sequences, rather than intact cells. The innovation of the method lies in the use of specially designed microarrays, onto which the DNA is hybridized with fluorescent PNA probes. Because each individual telomeric fragment becomes immobilized on the chip and is detected as a discrete fluorescent signal, the method achieves single-molecule resolution. High-resolution fluorescence microscopy is then used to capture images and quantify signal intensity, which is directly proportional to the TL.

Absolute TL can be determined by calibrating fluorescence intensity against a standard curve generated from telomeric sequences of known length. The integration of microarray platforms has markedly increased the throughput of FISH-based assays: from a single DNA sample, approximately 32,000 individual TL measurements can be obtained, while the array architecture allows simultaneous analysis of up to 96 samples per slide. The method is capable of detecting TL ranging from approximately 0.2 kb–340 kb. Another major advantage is its high analytical accuracy and excellent reproducibility (Table 1).

#### 2.2.4 Optical mapping

Optical mapping is a technology for analyzing long biopolymers, such as DNA, through fluorescent labeling of specific sequences, linearization of DNA on specialized chips, and subsequent visualization by fluorescence microscopy (Uppuluri et al., 2021). This method has been applied to measure TL, enabling haplotype-resolved analysis of telomeres at a single-molecule resolution (McCaffrey et al., 2017). The labeling process, known as nick translation, involves introducing single-strand nicks at telomeric and subtelomeric regions with specific nicking enzymes and Cas9n (Cas9 nickase). At these sites, Taq DNA polymerase, leveraging its 5'→3′exonuclease activity, excises nucleotides ahead and incorporates fluorescent nucleotides, while DNA ligase seals the breaks, restoring DNA integrity. The DNA is linearized within nanochannels on chips for analysis by fluorescence microscopy. This produces an almost continuous fluorescent signal from the telomeric DNA, with intensity proportional to TL. Comparison of fluorescent signals from subtelomeric regions with those from the reference genome enables identification of chromosome arms. Currently, bioinformatic tools such as TelOMpy are being developed to estimate individual TLs from optical genome mapping data (Pokrovac et al., 2025).

Optical mapping enables the measurement of very long telomeres (up to 100 kb) and has been successfully applied to differentiate ALT+ and TEL + cancer cells (Raseley et al., 2023). However, the method requires specialized equipment, including microfluidic chips, a high-resolution fluorescence microscope, and special software for data analysis. Thus, the technical complexity of this approach significantly limits its accessibility. Additionally, the incomplete nature of the reference genome has constrained the resolution of certain subtelomeric regions, notably the short arms of acrocentric chromosomes (13p, 14p, 15p, 21p, and 22p) (Uppuluri et al., 2021; Raseley et al., 2023). Comprehensive analysis of these challenging regions continues to depend on further optimization of analytical pipelines and methodological advancements.

#### 2.2.5 Telomere length combing assay (TCA)

TCA involves stretching DNA fibers uniformly on specialized glass coverslips, enabling direct visualization of the entire TL distribution. Telomeric regions are labeled with fluorescent PNA probes, and high-resolution fluorescence microscopy allows precise measurement of individual telomeric tracts in micrometers, which can then be converted to kb. To reduce DNA fragmentation, freshly isolated cells are embedded in agarose plugs, followed by protein digestion within the gel matrix. Results obtained with TCA display strong concordance with established methods—including TRF, qPCR, Q-FISH, and flow-FISH—with correlation coefficients *R* ^2^ ≈ 0.9 (Kahl et al., 2020). This approach is particularly suited for assessing long telomeres and has been instrumental in studying repair mechanisms within the ALT pathway (Broderick et al., 2023). Nonetheless, its reliance on specialized equipment, reagents, and sophisticated image analysis software, coupled with costs approximately twice those of flow-FISH, has limited its broader application.

#### 2.2.6 Single-molecule terminal restriction fragment analysis (smTRF)

In 2025, smTRF assay was developed, allowing direct measurement of individual TL, albeit without providing information on their chromosomal localization (Gao et al., 2025). Telomeric fragments are generated by digesting genomic DNA with a combination of restriction endonucleases (*Bfa*I, *Cvi*AII, *Mse*I, and *Nde*I), which produce sticky TA and AT overhangs at the 5′ ends of the resulting fragments. These ends are subsequently filled in with DIG-UTP and ATP, enabling immobilization of labeled fragments on glass surfaces coated with anti-DIG antibodies. The fragments are then hybridized with biotinylated oligonucleotides complementary to the telomeric 3′-overhang, followed by conjugation with streptavidin-coated magnetic beads. TL can be measured by single-molecule force spectroscopy employing magnetic tweezers, a technique that manipulates magnetic beads via a magnetic field gradient. The measurement is derived from analyzing the DNA stretching curve, which reflects molecular elongation under applied force. This approach achieves near–base-pair resolution.

A key advantage of this method is its applicability for studying the interaction of telomeric DNA with proteins. Magnetic tweezers enable real-time tracking of protein association and dissociation dynamics, as well as determination of binding energies. However, the technical complexity of the method and the need for specialized instrumentation restrict its widespread implementation.

#### 2.2.7 Mass *in situ* hybridization (MISH)

MISH is another recently proposed approach for measuring TL (Yan et al., 2025). In contrast to conventional FISH, which detects telomeric probes via fluorescence, MISH utilizes branched oligonucleotide structures labeled with holmium ions, with signals recorded through mass cytometry. Similar to flow-FISH, this method enables simultaneous assessment of telomeric signals and cell phenotypes, as mass cytometry permits parallel measurement of multiple markers labeled with distinct metal isotopes on both the cell surface and intracellular compartments. Phenotyping is conducted using panels of marker-specific antibodies, each conjugated to a rare-earth metal isotope.

Currently, MISH remains largely conceptual; quantitative TL analysis using this technique has yet to be realized due to the lack of standardized calibration protocols, technical challenges related to probe hybridization, and insufficient data for developing robust quantitative algorithms.

## 3 Methods based on hybridization and PCR

### 3.1 Single telomere length analysis (STELA)

**FIGURE 3:**
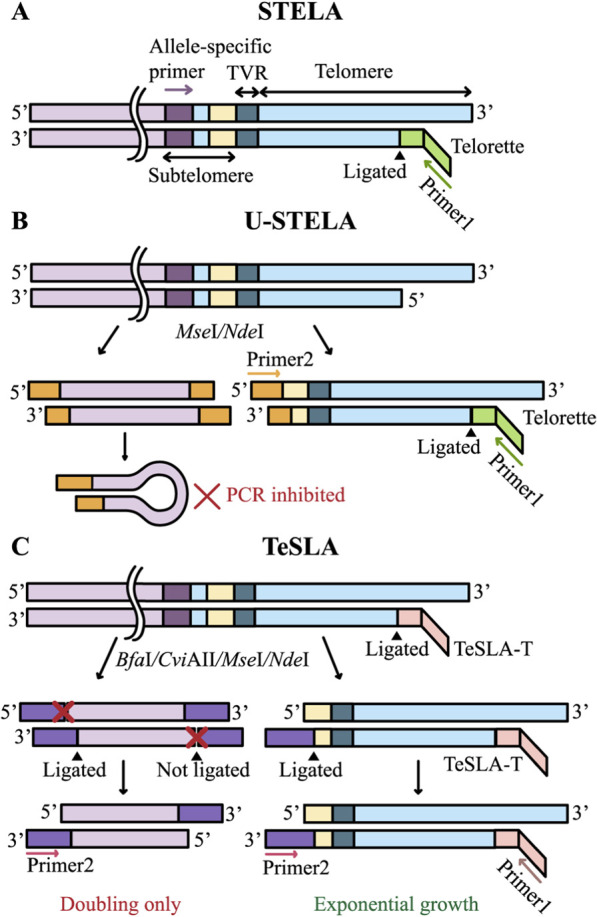
PCR in STELA, U-STELA, and TeSLA. PCR is followed by Southern blotting in all three methods. **(A)** STELA: the Telorette adapter is ligated to the telomeric C-rich strand, followed by allele-specific PCR amplification. **(B)** U-STELA: in addition to the Telorette ligation, genomic DNA is digested with *Mse*I and *Nde*I restriction endonucleases, generating sticky ends to which a double-stranded adapter (shown in orange) is ligated; this adapter serves as a PCR primer 2 binding site. When a double-stranded adapter is ligated to both ends of a DNA fragment, it forms a loop structure that effectively suppresses the amplification of internal genomic fragments. **(C)** TeSLA: genomic DNA is first ligated with TeSLA-T adapters, which serve as binding sites for the first PCR primer. The DNA is then digested with a cocktail of restriction enzymes (*Bfa*I, *Cvi*AII, *Mse*I, *Nde*I), followed by ligation of a second type of adapter (shown in purple) bearing a modified 3′-end that permits ligation of only a single strand. A second PCR primer complementary to these adapters is then employed. During amplification, non-telomeric fragments undergo linear replication, whereas telomeric fragments are amplified exponentially.

STELA was developed to measure TL at specific chromosomal ends (Baird et al., 2003). This technique involves ligating a specialized adapter to the telomeric DNA terminus, followed by allele-specific PCR amplification and analysis of the resulting telomeric fragments lengths (Figure 3A). STELA requires only minimal DNA input, typically in the picogram range, within the testing mix (Baird et al., 2003). Initially, genomic DNA extracted from cells is annealed with synthetic Telorette oligonucleotides, which contain a sequence complementary to the G-rich strand of telomeric DNA and a non-complementary 5′overhang that serves as a priming site for PCR. Subsequently, these annealed adapters are ligated to the C-rich strand of the telomere. PCR amplification is then performed using two primers: one complementary to the adapter sequence and the other specific to a unique subtelomeric sequence of a specific chromosome, ensuring the method’s chromosome specificity. To minimize artifacts, amplification is performed only to sub-visible levels, after which the products are separated by gel electrophoresis and analyzed via Southern blotting using a telomeric probe. A high-throughput version of this method, named HT-STELA, has been developed to eliminate the need for Southern blotting. In HT-STELA, PCR fragments are separated by capillary gel electrophoresis and detected through the fluorescence of an intercalating dye bound to double-stranded DNA (Norris et al., 2021).

STELA provides highly accurate measurements of TL and is particularly well suited for detecting critically short telomeres. It has enabled the discovery of ultrashort telomeres in senescent cells and revealed their allelic heterogeneity (Baird et al., 2003). Furthermore, it uncovered the roles of telomere critical shortening, dysfunction, and fusion in leukemia progression (Lin et al., 2010) as well as pronounced TL heterogeneity in placental tissue (Garcia-Martin et al., 2017). Nonetheless, STELA has notable limitations. It requires the presence of unique subtelomeric sequences located sufficiently close to the telomere to permit PCR. Because many chromosomes lack such proximal unique sequences, the applicability of STELA is limited. Reported applications include chromosomes XpYp (Baird et al., 2003; Britt-Compton et al., 2006), 2p, 7q (Norris et al., 2019), 11q, 12q (Baird et al., 2003; Britt-Compton et al., 2006), 17p (Norris et al., 2021), 21q (Ngo et al., 2021). Besides, STELA and its derivatives, U-STELA (Bendix et al., 2010) and TeSLA (Lai et al., 2017), rely on long-range PCR, which requires a high level of technical expertise and prevents the analysis of telomeres longer than ∼20 kb, thereby limiting broader applicability of these methods (Baird et al., 2003).

### 3.2 Universal STELA (U-STELA)

U-STELA is a modification of STELA designed to identify all short telomeres, but without determining their chromosomal localization (Bendix et al., 2010). Purified DNA is first digested with restriction endonucleases that generate sticky ends in subtelomeric regions. Subsequently, a double-stranded adapter containing a PCR primer sequence is ligated to these ends. Non-telomeric fragments carrying adapters at both ends form panhandle structures during PCR, thereby suppressing their amplification (Figure 3B). Similar to STELA, the Telorette adapters are ligated to the distal ends of telomeric DNA. Unlike STELA, this approach is aimed at the analysis of all chromosomes. However, it is not suitable for assessing mean TL because longer telomeres are amplified less efficiently and are therefore underrepresented in the results. In addition, amplification of ITSs is not fully suppressed, as the panhandle PCR suppression mechanism is primarily effective for short DNA fragments (Lavrentieva et al., 1999). Finally, amplified fragments include a portion of subtelomeric DNA of unknown length. These factors, together with the technical complexity of the protocol, have limited the widespread adoption of U-STELA.

### 3.3 Telomere shortest length assay (TeSLA)

TeSLA is an optimized adaptation of U-STELA designed to overcome its inherent limitations (Lai et al., 2017). In U-STELA, nonspecific ligation between subtelomeric sequences and restriction fragments of genomic DNA, together with nonspecific annealing of Telorette adapters, compromised assay specificity. TeSLA mitigates these issues by first ligating TeSLA-T adapters to genomic DNA prior to restriction digestion, followed by 5′-phosphatase treatment (Figure 3C). Subsequently, double-stranded adapters are introduced, but a terminal spacer at their 3′ends restricts ligation to a single strand. This configuration ensures that PCR amplification is confined to telomeric sequences, thereby substantially enhancing both the sensitivity and the specificity of TL measurement (Lai et al., 2017).

STELA, U-STELA, and TeSLA all enable the measurement of individual TL and were the only alternatives to Q-FISH prior to the advent of long-read sequencing technologies. However, their labor-intensive protocols and the requirement for advanced expertise in amplifying long DNA fragments and performing Southern blotting have limited their widespread adoption.

## 4 PCR-based methods

### 4.1 Quantitative PCR (qPCR)

qPCR is among the most widely used methods for TL assessment and remains the preferred technique for epidemiological and population-based studies due to its high throughput, cost-effectiveness, relative simplicity, and low DNA input requirement (typically 10–40 ng per reaction). Depending on the specific protocol, qPCR can be used to estimate either relative or absolute average TL. The method quantifies telomeric repeat amplification relative to a single-copy reference gene (for example, 36B4, albumin, or β-globin). Relative TL is expressed as the ratio of telomeric to reference signal intensity (T/S ratio).

In its original form (singleplex qPCR), telomere and reference gene amplification are performed in separate tubes (Cawthon, 2002). However, TL values obtained with this approach showed only moderate correlation with TRF analysis (*R* ^2^ ≈ 0.20–0.68) (Gutierrez-Rodrigues et al., 2014). To address this limitation, a modified protocol termed monochrome multiplex qPCR (MM-qPCR) was introduced, in which both amplifications are carried out in the same tube. Different melting temperatures of the amplification products permit separate quantification of the signals. In this version, telomeric primers generate a product of fixed length, unlike the earlier design that produced a heterogeneous set of amplicons (Cawthon, 2009). This modification reduced the number of reactions, improved cost-effectiveness, and, most importantly, enhanced accuracy, yielding stronger correlation with TRF (*R* ^2^ ≈ 0.84) and improved reproducibility across runs (*R* ^2^ ≈ 0.91) (Cawthon, 2009).

A method for absolute TL measurement was proposed based on synthetic oligomeric standards for both telomeric repeats and the reference gene. Calibration curves generated from serial dilutions of the standards allow quantification of the total telomeric DNA content in a sample (expressed in kb per reaction). This value is then normalized to the number of diploid genomes, determined from the single-copy gene, and divided by 92 (the total number of telomeres in the diploid human genome) yielding the average TL (O’Callaghan et al., 2008).

Currently, qPCR-based TL measurement remains the most commonly applied technique. By including a sample with a known TL, relative TL values obtained by qPCR can be converted into absolute average lengths without the use of calibration curves. Nevertheless, the method exhibits substantial variability, including discrepancies in data between various laboratories (Martin-Ruiz et al., 2015) (Table 1). This variability arises from differences in DNA input between replicates, choice of reference genes, DNA quality and integrity, and other technical factors (Lin et al., 2019; Tournoy et al., 2025). When analyzing tumor samples, the method may yield inaccurate results, as aneuploidy can lead to duplication or loss of the single-copy control gene, thereby biasing the T/S ratio (Holland and Cleveland, 2009). Employing primers to multiple-copy sequences сan partially mitigate this limitation (Dahlgren et al., 2018).

### 4.2 STAR (single telomere absolute-length rapid assay)

The STAR assay enables absolute measurement of individual TL using digital PCR (Luo et al., 2020). Genomic DNA is digested with restriction endonucleases that do not cut within telomeric repeats. The DNA fragments are then partitioned into numerous individual digital PCR reactions using a limiting dilution approach, originally proposed in (Lukyanov et al., 1996), ensuring that most reactions contain either a single telomeric DNA molecule or none at all. Amplification is performed using telomere-specific primers, and a set of calibration standards is employed to calculate the absolute TL. Unlike conventional PCR methods, STAR not only measures average TL but also generates a complete TL distribution within the cell population, including critically short and very long telomeres, though it does not provide information about chromosomal localization. The method shows a high correlation with TRF (*R* ^2^ ≈ 0.96). Importantly, because STAR does not rely on a single-copy reference gene for normalization, it remains accurate for aneuploid samples. Limitations of the method include the need for expensive equipment and skills in digital PCR techniques, as well as the necessity of optimizing reaction conditions for efficient performance (Luo et al., 2020).

### 4.3 qPCR-based TL analysis in single cells

The qPCR technology for TL measurement has also been implemented in a single-cell version, termed Single-Cell Telomere-Preamplification Quantitative PCR (SCT-pqPCR) (Wang et al., 2013). In this method, DNA is extracted from individual cells and subjected to preamplification to obtain sufficient material for analysis. qPCR is then performed, concurrently amplifying telomeric sequences and a reference locus. Notably, single-copy genes frequently produced unstable results and multi-peak melting curves, suggesting their poor amplification under such conditions. By contrast, multicopy loci (for example, Alu repeats) yielded more reproducible results. SCT-pqPCR revealed that TL varies substantially among individual cells within a single population (CV 17%–49%). Cells from elderly donors and late-passage cultures exhibited higher variability, and cancer cell lines showed greater intercellular heterogeneity in TL compared to normal cells. A limitation of the method, beyond its technical complexity, is the inability to evaluate potential artifacts introduced during the preamplification step (Wang et al., 2013).

ΩqPCR is another single-cell based approach that, unlike SCT-pqPCR, does not require a preamplification step (Xiong and Frasch, 2021). Instead, a sophisticated set of specialized Ω-probes is used, which hybridize to the telomeric repeats of genomic DNA isolated from a single cell. Hybridization is performed slowly and in the presence of an excess of probes to ensure complete coverage of the entire telomeres. Only those Ω-probes that have fully hybridized to telomeric DNA can be subsequently ligated, resulting in the formation of circular DNA structures. The Ω-probes are designed so that only ligated probes can be amplified. Each probe covers a fixed number of nucleotides within the telomeric sequence, and collectively, the probes provide complete coverage of the telomeric DNA. Consequently, TL is directly proportional to the number of circularized probes that undergo amplification. This method enables the estimation of the mean TL per cell.

The technical complexity of both SCT-pqPCR and ΩqPCR currently limits their widespread application. Nevertheless, their emergence highlights the growing demand for single-cell TL analysis and suggests that more accurate and accessible methods are expected to emerge in the near future.

## 5 Sequencing technologies

### 5.1 Short-read sequencing

Next-generation sequencing (NGS) technologies rely on reading short DNA fragments (typically 50–300 base pairs) and allow estimation of the mean TL in a sample by quantifying telomeric repeats within sequencing reads. TL estimation via NGS is particularly advantageous when whole genome sequencing (WGS) data have already been generated for other purposes, as TL calculation can then be performed solely through bioinformatic analysis of existing data. Nevertheless, this approach has several important limitations. First, short-read sequencing allows estimation of only the mean TL across the entire genome. Moreover, because telomeres are composed of microsatellites and read lengths are far shorter than the telomere itself, aligning telomeric reads to the reference genome is challenging. Further complications arise from ITSs scattered throughout the genome, which can produce artefactual signals that mimic true telomeres. Several bioinformatic tools have been developed to analyze WGS data for TL assessment, including Motif_counter (Conomos et al., 2012), TelomereHunter (Feuerbach et al., 2019), TelSeq (Ding et al., 2014), Computel (Nersisyan and Arakelyan, 2015), and TelomereCat (Farmery et al., 2018) and have been validated using Illumina-based sequencing data. These tools require FASTQ or BAM files as input, making them suitable for bioinformatic processing of data generated by most NGS platforms.

Motif_counter (Conomos et al., 2012) and TelomereHunter (Feuerbach et al., 2019) are designed to estimate the relative abundance of telomeric sequences. The methods enable users to define the motif used for telomeric read identification, the required number of repeats, and whether the repeats must be consecutive or not. The tools operate on sequence reads aligned to a reference genome and stored in Binary Alignment/Map (BAM) format. The output reports telomere content as the number of sequencing reads containing telomeric repeats. However, the more recent tool TelomereHunter outperforms Motif_counter by enabling robust comparison across samples with varying sequencing depths and applying correction for sequencing bias related to GC content and effectively analyzing variations of the canonical telomeric hexamer, thereby detecting non-canonical repeats. Thus, Motif_counter and TelomereHunter together provide quantitative and qualitative insights into telomeric sequence composition but do not directly measure TL. These methods are applied primarily in large cancer genome cohort analyses and for the identification of samples with ALT-phenotypes (Lee et al., 2014; Cornish et al., 2024).

TelSeq (Ding et al., 2014) and Computel (Nersisyan and Arakelyan, 2015) enable estimation of mean TL assuming a known chromosome number per cell, limiting their applicability for analyzing cancer samples. Specifically, TelSeq estimates TL by identifying reads containing seven or more TTAGGG repeats from aligned BAM reads. A distinctive feature of TelSeq is its original correction for GC-content-dependent sequencing bias, which affects short-read data: read coverage is elevated in GC-rich regions (Dohm et al., 2008). TelSeq normalizes the telomeric read count by the proportion of total reads with a GC content between 48% and 52%, chosen to approximate the GC composition of telomeric sequences, which substantially improves correlation with TRF (Ding et al., 2014). Additionally, TelSeq is simple to use, which represents a significant advantage. (Lee et al., 2017).

Unlike the motif-search-based tools described above, Computel (Nersisyan and Arakelyan, 2015) aligns raw fastq reads to a specially generated telomeric reference, thereby enabling the filtering of ITSs. The number of telomeric reads obtained is normalized to genome coverage after alignment to the reference genome, and the mean TL is then calculated. (Nersisyan and Arakelyan, 2015).

The TelomereCat (Farmery et al., 2018) is free from assumptions about chromosome number per cell. The method is based on the ratio between reads that fully cover telomeric sequences and reads that span the junction with subtelomeric regions. In addition, TelomereCat accounts for the contribution of ITS reads using an approach based on the assumption of symmetric distribution of reads at ITS boundaries: since ITS are flanked on both sides by non-telomeric sequences, reads spanning ITS junctions are equally likely to map to the 5′or 3′end of the ITS. By contrast, true telomeres are located at chromosome ends and have a boundary on only one side, which produces an asymmetric distribution of junction-spanning reads. By comparing the frequencies of these reads, TelomereCat can estimate and exclude the ITS contribution without requiring precise alignment of these difficult-to-map regions (Farmery et al., 2018). Nevertheless, the correlation of this method with TRF and qPCR is weaker than that achieved by Motif_counter, TelomereHunter, TelSeq and Computel (Feuerbach et al., 2019).

Taken together, these methods provide a rapid assessment of the abundance of telomeric sequences or TL and show good correlation with qPCR results. Their correlation with TRF is weaker, which may be due to the inclusion of subtelomeric regions in TRF analysis outputs.

### 5.2 Long-read sequencing

In recent years, the rapid advancements in long-read sequencing platforms by Oxford Nanopore Technologies (ONT) and Pacific Biosciences (PacBio) has facilitated their application to TL measurement. With average read lengths of 10–30 kb (and occasionally exceeding 100 kb), this approach now enables the generation of TL profiles for individual chromosome ends. Thus, long-read sequencing has become the first method capable of determining both the nucleotide composition and the length of all telomeres in a sample with single-nucleotide resolution (Sholes et al., 2022; Tham et al., 2023; Schmidt et al., 2024; Karimian et al., 2024; Sanchez et al., 2024; Theulot et al., 2025). Moreover, the availability of complete T2T assemblies provides high-quality reference genomes for this purpose, enabling chromosome arm- and allele-specific telomere localization (Nurk et al., 2022; Liao et al., 2023). The single-nucleotide resolution of these T2T assemblies enabled the discovery of an unusual TL regulation mechanism in model organisms, based on the addition of non-telomeric dT residues to the chromosome’s 3′end, which may help elucidate analogous regulatory processes in humans (Eremin et al., 2025).

In practice, the lower-cost and more widely adopted ONT technology is primarily used for TL measurement, whereas the application of PacBio, despite its higher accuracy, remains limited. This limitation is also due to the shorter read lengths typically generated by PacBio, which often fail to provide sufficient resolution for chromosome-specific analysis, as well as the requirement for DNA restriction digestion. The latter introduces an additional step and a challenge, as restriction enzymes must be carefully selected to avoid cutting within telomeric tracts while preserving sufficiently long subtelomeric regions to enable chromosome-specific mapping (Tham et al., 2023).

**FIGURE 4:**
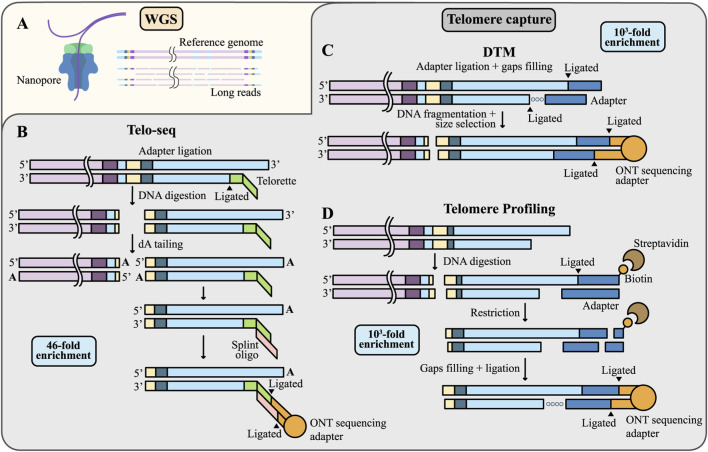
TL measurement using WGS or telomere enrichment techniques. **(A)** The proportion of reads containing telomeric sequences is very low in WGS data. **(B**–**D)** Strategies for telomeric reads enrichment. **(B)** In Telo-seq, the Telorette adapters are annealed to the G-rich telomeric strands and ligated to the C-rich strands. Genomic DNA is then digested with the blunt-end restriction endonuclease, followed by addition of dA-tails to the resulting ends to prevent concatemer formation. A splint oligonucleotide is annealed to the Telorette adapter and then ligated to a sequencing adapter with a motor protein; **(C)** In DTM, double-stranded adapters (shown in blue) are ligated to the 3′overhang of telomeric DNA, gaps on the C-rich strand are filled by DNA polymerase (dotted line), then the DNA is mechanically fragmented and sequencing adapters are ligated; **(D)** In Telomere Profiling, genomic DNA is digested and telomeric ends are ligated to biotinylated double-stranded adapters (shown in blue). Labeled fragments are captured with streptavidin beads, enzymatically released, and ligated to sequencing adapters. Note that methods **(C,D)** include the 3′telomeric overhang in the output sequencing data.

Notably, telomeric regions comprise only about 0.01% of the human genome, resulting in their extremely limited representation within standard WGS datasets (Figure 4A) (Karimian et al., 2024). To overcome this, strategies for telomere read enrichment are being actively developed. The first such strategy was introduced in 2023 for PacBio technology (Tham et al., 2023), and three distinct approaches for ONT sequencing were launched in 2024, reflecting the strong demand for these methods: Telo-seq (Schmidt et al., 2024), Digital telomere measurement (DTM) (Sanchez et al., 2024), and Telomere Profiling (Karimian et al., 2024). All methods involve ligating sequencing adapters directly to telomeres and digesting genomic DNA to remove non-telomeric fragments (Figures 4B–D). This approach markedly increases the fraction of telomeric reads, thereby reducing costs and improving the accuracy of TL estimation. Telo-seq yields approximately a 46-fold enrichment of telomeric reads compared to WGS (Schmidt et al., 2024), while both DTM and Telomere Profiling achieve around a 1000-fold enrichment (Karimian et al., 2024; Sanchez et al., 2024). The detailed and updated Telo-seq protocol is currently available on the ONT website. All methods enable multiplexing, allowing the recovery of up to approximately 50,000 telomeric reads per flow cell, with an average fragment length of ∼20 kb for ONT (including both subtelomeric and telomeric sequences). The estimated cost per sample with multiplexing ranges from approximately 80–140 USD (Karimian et al., 2024).

Together with advances in telomeric enrichment techniques, bioinformatic tools for the analysis of telomeric reads have been developed. These tools facilitate the isolation and assembly of telomere-containing reads and the identification of telomere–subtelomere boundaries, typically through windowing approaches, thereby enabling TL determination. When reference genomes are available, these methods allow for chromosome arm- and allele-specific TL estimation. Furthermore, tools designed to correct basecalling errors within telomeric regions have recently been introduced (Karimian et al., 2024). Particularly noteworthy are the tools developed following the T2T human genome assemblies, which leverage the most complete reference sequences to yield highly precise results.

**FIGURE 5:**
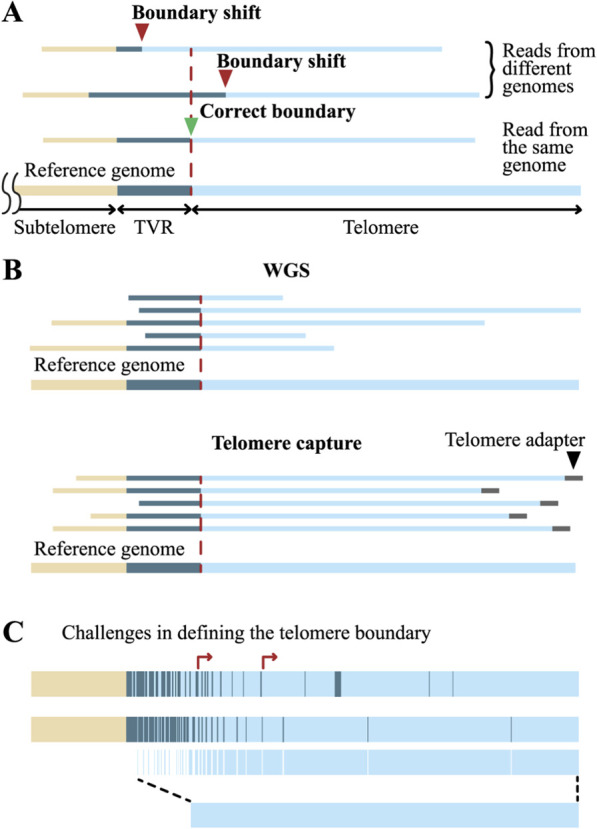
Challenges in estimating TL from long-read sequencing data. **(A)** Alignment of reads to a non-identical reference genome may lead to inaccurate determination of the telomere boundary. **(B)** Telomere capture techniques allow selection of only full-length telomeric reads and enable more accurate TL measurement. **(С)** The complexity of TVR patterns hampers precise delineation of the proximal telomere boundary.

Long read sequencing data enable TL determination by two approaches: reference-based mapping and reference-free methods. Reference-based methods typically use T2T-CHM13 for chromosome-specific (Nurk et al., 2022) and phased HG002 genomes for allele-specific mapping (Garg et al., 2021). First, the telomere–subtelomere boundary is defined in the reference genome, then experimental reads are aligned to this reference. To improve precision, telomeric regions of the reference can be deleted or masked, retaining only subtelomeric regions (Karimian et al., 2024; Stephens and Kocher, 2024). TL is calculated from the first nucleotide of the telomeric read that does not align to the subtelomeric region in the reference genome and extends to the last nucleotide before the telomere capture adapter (or simply to the last nucleotide of the read in WGS). However, this approach works well only for genomes that are identical or highly similar (Karimian et al., 2024; Deimler et al., 2025). When mapping to a non-identical genome, due to variability in the length and composition of subtelomeric sequences, a substantial portion of reads may fail to align. Besides, the actual boundary between the telomere and subtelomere in the sample may not coincide with the boundary in the reference, resulting in over- or underestimation of TL (Figure 5A). In such cases, the human pangenome may sometimes be used (Liao et al., 2023), but it still does not capture the full diversity of alleles. Moreover, if alignment to the reference genome is set as a mandatory criterion for selecting telomeric reads, the allelic diversity present in the analyzed sample will be lost, as subtelomeric variants absent from reference genomes may exist in the sample. Therefore, in these cases, the telomere-subtelomere boundary is identified directly from the reads, and TL is measured from this boundary to the end of the telomeric sequence, or to the beginning of the adapter in enriched libraries.

Precise identification of the telomere’s start and end points is essential for any mapping method and technology of WGS. The distal, or external, end is relatively straightforward to determine: in WGS, it corresponds to read termini that end within the telomeric sequence, while in enriched libraries, it corresponds to reads terminating at capture adapters. Except for Telo-seq, all enrichment methods include the 3′overhang tail in the TL measurement (Figures 4B–D). Importantly, when using WGS, distinguishing truncated telomeric reads from full-length ones is not possible, whereas in enriched libraries, the presence of capture adapters on full-length reads allows truncated reads to be excluded from the analysis. Consequently, TL determined from WGS reads may be underestimated (Figure 5B).

The determination of the internal, proximal boundary of the telomere is considerably more complex. Apart from the possible lack of a clear boundary between the telomere and TVR region, the main challenge lies specifically within the TVRs themselves. These degenerate telomeric repeats exhibit diverse structural variants, primarily comprising single-nucleotide substitutions and indels, with the lengths of TVR regions typically ranging from 0 to 2 kb, although in some alleles they may extend up to 8 kb (Figure 1B) (Tham et al., 2023; Stephens and Kocher, 2024). Although TVRs density generally drops sharply at the TVR region boundary, transitioning into canonical telomeric repeats, TVR stretches and isolated repeats are dispersed within the telomere outside the core TVR region, with densities varying significantly across chromosomes and individuals (Tham et al., 2023; Karimian et al., 2024; Stephens and Kocher, 2024). This heterogeneity substantially contributes to the complexity of delineating the proximal telomere boundary (Figure 5C).

**FIGURE 6:**
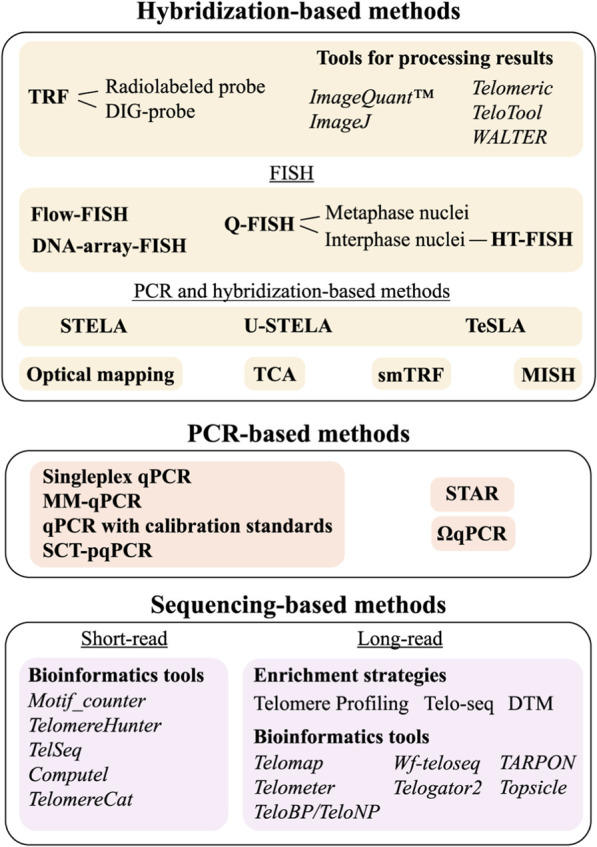
TL measurement methods classified according to the core technique used. See text for details.

Several algorithms have recently been developed to determine TL from long-read sequencing data (summarized in Figure 6), employing diverse strategies for identifying the proximal telomere boundary (Tham et al., 2023; Karimian et al., 2024; Sanchez et al., 2024; Stephens and Kocher, 2024; Deimler et al., 2025; Nguyen and Choi, 2025). The most straightforward approach is implemented in the Telomap algorithm (Tham et al., 2023), designed for PacBio reads, and in Telometer (Sanchez et al., 2024), which builds upon Telomap and is adapted for ONT data. In these algorithms, the telomere boundary is defined by the presence of two consecutive canonical TTAGGG motifs, based on the observation that the minimal shelterin-binding site spans approximately 1.5 telomeric repeats (König et al., 1998; Bianchi et al., 1999; Hanaoka et al., 2005). Alternatively, TVRs can be excluded from TL calculations (Tham et al., 2023), based on their impaired capacity to bind shelterin complex (Broccoli et al., 1997; Nishikawa et al., 2001; Conomos et al., 2012) (Figure 5C). A more sophisticated approach is implemented in the TeloBP algorithm: the chromosome is scanned from the end inward using a rolling window, and the telomere boundary is identified by detecting a discontinuity in a pattern in which at least 50% of the nucleotides are GGG. This pattern can be modified by the user (Karimian et al., 2024). A similar strategy is employed in the Telogator2 algorithm, where the boundary is determined based on the density of telomeric repeats within a sliding window; however, the set of telomeric repeats also includes approximately ten TVR variants, most of which contain the CCC pattern (Stephens et al., 2022). In the Topsicle algorithm proposed in 2025, which is designed to measure TL in non-enriched WGS libraries from various organisms, the telomere boundary is determined by detecting a window exhibiting a pronounced decrease in telomeric repeat density using change-point detection analysis (Nguyen and Choi, 2025). In the TARPON pipeline introduced the same year, the entire TVR region is incorporated into the telomeric sequence during TL calculation (Deimler et al., 2025), whereas in the wf-teloseq algorithm, a component of the ONT Telo-seq workflow, all common erroneous telomeric patterns arising from basecalling artifacts (see below) are treated as canonical repeats (Schmidt et al., 2024). It should be noted that TARPON and wf-teloseq differ favorably from other algorithms in that they are available not only as source code, which requires users to possess advanced bioinformatics skills, but are also integrated into ONT’s EPI2ME agent, providing a user-friendly graphical interface. Clearly, the proximal telomere boundary defined by all these methods may vary, with discrepancies reaching several hundred base pairs (Tham et al., 2023; Karimian et al., 2024). Given that long-read sequencing enables TL determination with nucleotide-level precision, such variation is rather substantial. Emerging comparative studies of these methods are anticipated to help identify optimal analytical strategies and foster consensus on their application (Deimler et al., 2025; Reed et al., 2025).

The challenge of determining TL is further complicated by the high frequency of basecalling errors in telomeric regions in ONT data. For example, the canonical telomeric repeat TTAGGG is frequently miscalled as TTAAAA, which can lead to overestimation of the fraction of TVRs or even misclassification of telomeric regions as subtelomeric (Tan et al., 2022). Several recurrent error patterns have been identified, and various correction approaches have been developed to address them (Karimian et al., 2024; Liu et al., 2024). For example, Karimian et al. developed the TeloNP algorithm, which corrects ONT basecalling errors by leveraging these recurrent error patterns, allowing users to adjust them to accommodate common new errors generated by updated basecallers. They also introduced TeloPeakCounter, which counts repeated waves in the raw electrical signal output of the MinION and is based on the finding that peak counting provides higher accuracy than basecalled sequences in repetitive DNA regions (Karimian et al., 2024). Currently, ONT has incorporated improved telomere basecalling into its default Dorado basecaller.

While ONT currently offers reads of sufficient length for TL determination, basecalling errors impede precise assessment of TVR distribution. At the same time, PacBio’s higher accuracy enables precise telomeric sequence recovery (Liu et al., 2024), but the reads are often insufficiently long to fully span all telomeres and unambiguously assign them to specific chromosomes (Tham et al., 2023; Sanchez et al., 2024). Neither platform currently supports measurement of very long telomeres due to read length limitations. Nevertheless, despite these obstacles, long-read sequencing remains the only method capable of determining chromosome- and allele-specific TL with nucleotide-level resolution. Moreover, it is uniquely suited for *de novo* telomere characterization, enabling discovery of novel alleles and distinct TVR distribution patterns. Long-read sequencing has been successfully applied to measure TL across various cell lines, in humans, and other organisms (Karimian et al., 2024; Sanchez et al., 2024; Nguyen and Choi, 2025); its results correlate strongly with TRF and Q-FISH measurements (Sanchez et al., 2024). The method is applicable in both large-scale epidemiological and longitudinal studies of telomere shortening in individuals (Tham et al., 2023).

Long-read sequencing enables not only the measurement of allele-specific TL. The Nanotiming technology, introduced in 2025, enables determination of the replication timing of individual telomeres during the S-phase using long-read sequencing. The method relies on the incorporation of 5-bromodeoxyuridine (BrdU), a thymidine analogue, into DNA during replication. Since BrdU competes with endogenous dTTP, its incorporation varies depending on replication timing: at the beginning of the S-phase, when dTTP concentration is low, BrdU is incorporated actively; during later stages, incorporation decreases. Thus, BrdU content in specific DNA regions serves as a marker of their replication timing. Nanotiming allows not only replication timing assessment but also simultaneous measurement of TL at individual chromosome ends (Theulot et al., 2025).

## 6 Conclusion and future directions

To date, approximately two dozen methods are available for the measurement of TL (Figure 6), reflecting both the high demand for such analyses and the inherent complexity of the task. No single method currently combines accuracy, affordability, and ease of use. This complexity is further heightened by the pronounced heterogeneity of TL across different cell types, chromosomes, and even between homologous chromosomes. Consequently, researchers have to carefully select the most suitable method for their specific aims. Among the numerous approaches, only a few are widely adopted (Table 1), simplifying method selection. Mean TL is most commonly determined using TRF, qPCR, and Flow-FISH methods, while the shortest telomeres are measured using Q-FISH, STELA, and TeSLA. It is important to note that some methods may include sequences beyond the actual telomere in their TL measurements: TRF analysis typically encompasses the TVR region and part of the subtelomeric region, which can lead to an overestimation of TL by several kb. STELA results also include TVR region and subtelomeric segments; however, since the primer location is known, these regions can be subtracted from the final measurement. Although FISH probes are specific to telomeric repeats, it is not possible to fully exclude their hybridization with TVRs. The most specific measurements of true TL are obtained by qPCR and sequencing, with long-read sequencing providing chromosome-specific TL information. For convenience, a comparison of the existing methods, including their key features, is summarized in Table 1.

In practice, the choice of method is largely dictated by the scale of the study. For large cohort studies requiring high throughput, qPCR remains the preferred method. However, despite its widespread use, qPCR suffers from limited accuracy and reproducibility, and it does not provide information on TL distribution within a sample or the presence of critically short telomeres. Importantly, it is the shortening of individual telomeres, rather than a decrease in mean TL, that is believed to trigger cellular senescence and pathological processes (Kimura et al., 2010a; Dhabhar et al., 2012). Consequently, qPCR-based results allow the establishment of statistical associations between average TL and specific diseases but do not provide evidence for causal relationships. Nevertheless, most epidemiological studies relating TL to age, disease, and lifestyle factors have employed this approach and continue to do so. Currently, long-read sequencing offers the most precise TL measurements, but its high cost and limited throughput restrict widespread adoption. However, ongoing technological advancements in long-read sequencing are expected to reduce costs and improve performance, as has been observed with earlier sequencing methods. It is noteworthy that several telomere enrichment techniques (Karimian et al., 2024; Sanchez et al., 2024; Schmidt et al., 2024) and specialized data analysis pipelines (Karimian et al., 2024; Sanchez et al., 2024; Stephens and Kocher, 2024; Deimler et al., 2025; Nguyen and Choi, 2025) have been introduced in the past 2 years, already making telomere sequencing more affordable and faster. When combined with measurements of other biomarkers, long-read sequencing-based TL analysis has the potential to greatly enhance the accuracy of large-scale studies and provide insights into the mechanisms linking TL with aging and age-related diseases.

Long-read sequencing has finally enabled chromosome arm- and allele-specific TL measurement, but has revealed new challenges: it turned out that TVRs can be dispersed within telomeric sequences, existing both as stretches and single repeats among the canonical TTAGGG blocks along nearly the entire length of the telomere (Figure 5C). Moreover, their proportion and distribution patterns vary not only among chromosomes but also between individuals (Tham et al., 2023; Karimian et al., 2024; Stephens and Kocher, 2024). This raises several important questions. First, do these dispersed TVRs reduce TL measured by TRF, FISH, and PCR, and if so, to what extent? How can samples with identical TL but different proportions of dispersed TVRs be distinguished? Even more critically, how do the proportion and pattern of TVRs affect the protective functions of telomeres, considering that shelterin complex binding to TVRs is impaired (Tham et al., 2023)? Thus, telomere function may depend not only on its length but also on the balance between canonical and variant repeats, with TVR patterns modulating the detrimental effects of telomere shortening. Consequently, individual TVR patterns within telomeres may serve as novel biomarkers of susceptibility to age-related diseases and cancer. For instance, a longer telomere containing a high proportion of dispersed TVRs may provide less protection to a cell than a shorter telomere composed entirely of canonical repeats. Because TVR density peaks in the proximal telomeric regions, their impact on the protective function may increase as telomeres shorten. This may lead to an abrupt loss of cellular phenotype and create inter-individual variability, whereby telomeres of identical length confer unequal protection depending on their TVR composition.

Recent research on TL has raised several important questions in telomere biology and outlined key directions for future investigation: what molecular processes account for TL variability across chromosomes? Do all chromosomes shorten at comparable rates during cell divisions? On which chromosome(s) do critically short telomeres emerge first, and is this occurrence consistent or stochastic? Do genes located near the shortest telomeres contribute to detrimental cellular outcomes? Does the fate of a cell depend on which specific chromosome has become critically shortened? The current set of TL measurement methods, combined with other approaches, may provide answers to these questions. Furthermore, considering the heterogeneity of cell populations, future efforts will likely focus on single-cell TL analysis with dynamic tracking across successive divisions.

Another major challenge lies in the diversity of available techniques and methodological nuances, which complicate cross-study comparisons. Standardization efforts, similar to those developed for qPCR (International Organization for Standardization, 2019; Hays et al., 2024; Bustin et al., 2025), are therefore urgently needed. Initial steps have been taken by the Telomere Research Network (Lindrose and Drury, 2020), yet broader consensus and coordinated initiatives remain essential.

In summary, TL measurement provides an important contribution to understanding telomere biology and its relationship with age-related processes. Progress in this field is closely linked to advances in TL determination methodologies. Further refinement of these approaches, particularly long-read sequencing methods, will enable deeper insights into TL dynamics throughout life and during disease progression, elucidate the contributions of genetic, environmental, and lifestyle factors to TL variation, and support the development of therapeutic strategies aimed at treating age-related diseases and promoting healthy longevity.
